# Telling the Public about Medicine
*B.M.A. Popular Lecture: Bristol, Wednesday, 24th November, 1954.


**Published:** 1956-01

**Authors:** I. Harvey Flack

**Affiliations:** The Editor of "Family Doctor"


### TELLING THE PUBLIC ABOUT MEDICINE*

BY

I. HARVEY FLACK, M.D.

*The Editor of " Family Doctor "*

Ever since there were doctors they have had to consider the problem of telling the public about medicine. For many centuries it was not really a big problem. Medicine was mostly magic. It was a mixture of Babylonian numerology, Arabian alchemy, medieval horoscopy and inspired water-divining, or uromancy.

The profession disagreed about a lot of things. Whether seven was a better number than fourteen. Whether bleeding the patient when the moon was waxing was more effective than leeching when the moon was on the wane. But on one thing they were united. Medicine was a mystery—and the more mysterious it could be kept the better. The idea of taking the public into their confidence simply never occurred to them. They were engaged in the practice of magic. And the most amateur magician knows that the effect of his tricks disappears if the audience knows how they are done. So for a long time the orthodox professional view in this country was very much in favour of keeping the public in the dark.

Obviously when medicine was compounded more of myths and magic than of sense and science there were good reasons for this attitude. But as medicine became more rational and more scientific, the reasons for telling the public and winning their confidence were strengthened, and the reasons for preserving the aura of mystery had less and less force.

Today things have changed greatly. As a profession we have a great deal to tell the public. We have a more literate and better-informed public that really does want to know. And we have many more means of telling them. These are three recent and important changes—in the people at large, in methods of communication, and in medicine itself.

### BETTER-EDUCATED PUBLIC

Look first at the consumers of information, the ordinary people who create the demand for knowledge. They are better educated than ever before. The history of State education in England did not begin until 1832, but it has progressed rapidly. Today we are rather shocked when we learn that a mere 3 to 4 per cent. of Army recruits are " of very low literacy ". These Army figures are not far removed from those of a survey of reading ability undertaken in 1948 by the Ministry of Education. The yardstick was the ability to read as well as the average child of seven. Examining fifteen-year-olds on this basis, 1·4 per cent. were found to be illiterate and 4·3 per cent. semi-literate.

In other words we have nowadays a population which is 95 per cent. literate. Taking the simplest definition, they really can read and if they don't read what we as doctors write about medicine and health, they will read what somebody else writes.

### CHANGE IN THE PRESS

Now look at the change in the Press. The development of the popular press really took place in this present twentieth century. According to Herd (1952) the first news-book in this country was published on May 23rd, 1622. The first all-advertisement weekly was the *Publick Adviser* which appeared in 1657 and carried one of the earliest pieces of medical information ever to be seen in the public prints.

* B.M.A. Popular Lecture: Bristol, Wednesday, 24th November, 1954.

" In Bartholomew Lane on the backside of the Old Exchange, the drink called Coffee, (which is a very wholsom and physical drink, having many excellent vertues, closes the Orifice of the Stomack, fortifies the heat within, helpeth Digestion, quickneth the Spirits, maketh the heart lightsom, is good against Eye-sores, Coughs or Colds, Rhumes, Consumption, Head-ach, Dropsie, Gout, Scurvy, King's Evil and many others) is to be sold both in the morning and at three o'clock in the afternoon."

By 1711, according to a statement published by the Treasury, the total sales of all the papers then published amounted to 44,000 copies weekly, or 2¼ million copies in a year. Today the *Radio Times* and the *News of the World* both sell well over 8 million copies a week.

Almost exactly a hundred years ago *The Times* had a daily circulation of 60,000. And the second largest circulation, that of the *Morning Advertiser*, was only 6,600. Today *The Times* has a daily circulation around the quarter-million mark. There are now 5,723 newspapers and periodicals published regularly in the U.K., and for a big daily or a famous weekly a circulation of a million copies is a commonplace.

Broadcasting and television with their impact on millions of people are very much developments within our own lifetime. The pace of invention has speeded up greatly. So today there are more ways of telling people about medicine. There are more people who want to know, and who can read and understand. In this setting of increasing demand and improved and elaborate methods of communication, medicine has a great deal more to say for itself.

ADVANCES IN MEDICINE

I was a house-surgeon at the Manchester Royal Infirmary no more than twenty years ago. But let me give you a short-list of the developments since that time. We had no sulphonamides then. We had no penicillin, no antibiotics. Nobody had ever heard of radio-active isotopes. A blood transfusion was not a routine, but quite an event. Cardiac surgery was unknown and chest surgery was in its infancy. The tempo of scientific advance in medicine has quickened and accelerated right through this century. So let me stress again that we have more to tell, more people who want to know, and more and better means for telling them.

Now take the main question: What should we tell the public?

I think you can split up medical and health information into different levels. At the lowest level there is complete agreement in the profession. We all pay at least lip-service to health education. We want people to know about how the body works and why. We want them to know and profit by some of the nutritional knowledge gained over the last fifty years. There are all sorts of points about diet, exercise, fresh air, cleanliness, pasteurized milk, and moderation in, for example, the consumption of alcohol and tobacco, which we want people to learn. This is the basic level of what I would call pure health education, and it includes that instruction in the elements of biology and of human anatomy and physiology which is being taken up increasingly in the schools.

Now the next and more adult level of health education presupposes some of this knowledge and goes on to take a wide view of preventive medicine. It emphasizes the importance of clean food and of modern methods of inoculation and immunization. It includes, though not everybody approves of this, instruction on the early signs and symptoms of such diseases as cancer and tuberculosis.

Of course you can frighten people into cancerophobia. But if you conduct your education about cancer in the right way, and in the right setting you can only do good. I have yet to see any convincing evidence that the early detection of cancer has led to any increase in mortality.

Here there is some controversy but not all that much. Now take the next level of information and instruction and we come to what may be called the medical education of the public. This is the really controversial field. There are two questions here.

First. Should we instruct the public about new drugs, new treatments, new and old diseases?

Secondly. If we do so, then how far do we take the instruction and the argument?

I think the answer to the first question is simple enough. The public want to know about new drugs and new techniques. They love wonder-drugs and miracle cures. And if we don't take over the duty and responsibility of instruction—then someone else will.

You can really state the question in an alternative form. Is the public to receive responsible, authoritative and accurate instruction? Or is the instruction to be left to lay journalists, lay broadcasters—and the lunatic fringe of our own profession?

I feel that as a profession we have a real duty in this field. The days when medicine could be a mystery have long passed. For one thing we have too many intelligent laymen and laywomen inextricably mixed up with the most recent advances in medicine. Some recent figures are helpful. Today the Medical Research Council has a staff of 1,400. Of these 450 are " scientific staff " and only 159 of these are doctors. We need chemists and physicists and atomic scientists just as much as they need us. Whether we like it or not, we are treating a public which is better educated and better informed than was the case even twenty years ago.

Important, too, is the fact that we can use the demand for education about disease as one of the simplest and most acceptable modes of entry into the fields of health education. We must be realistic. We must accept the fact—however much some people may deplore it—that the public at large is not interested in health.

The few people who are passionately keen on pure health topics fall into two groups. There is a sane and converted group who are really interested in health education. There is a larger group of out-and-out cranks and faddists. They cling to nature cures or similar oddities and they buy the many magazines which are devoted to obscure " health cults ". I doubt if both groups add up to more than 1 per cent. of the population.

The ordinary man or woman in the street—the other 99 per cent.—is not interested in health. He or she is passionately interested in departures from health, in disease and its treatment. On any social occasion, nobody talks about health. Over the bridge table and in the intervals of canasta the talk is of my operation, my gynaecologist, my children's ailments. If, as a doctor, you go to a party you are never pestered by patients or potential patients only about health matters. What they want to know about is the latest and most fashionable disease, the new wonder drug, the latest operative miracle.

There is a real interest in disease and its treatment. And if you meet that interest and supply that demand for information, people will listen to you—or read what you write about it. They will go on listening to you—or go on reading—anything you may say or write in the same context which has to do with health education or preventive medicine in its widest sense. This I am sure is the most rewarding approach to the medical education of the public.

As a profession we must give them the information they seek about disease. We must take every opportunity while so doing to press home some of the relevant points about prevention, about early diagnosis, and about the accepted rules for healthy living. All this is the more important because on some of the problems of public health we have reached a point where we have most of the answers—and the public does not know them, or does not heed them.

### DIPHTHERIA

From 1930 to 1940 we had about 58,000 cases of diphtheria and some 3,000 deaths every year in this country. The propaganda campaign on this subject started in 1941 and by 1942 was well under way. In 1949 the case mortality was much the same as it had been ten years before. But the total number of notifications was

1,890 and there were 84 deaths. The figures for 1953 show 266 cases and 23 deaths. Diphtheria is now a rare cause of death.

This was not a triumph for the laboratory workers or the clinics. Their triumph was years ago. This successful campaign was a triumph for public medical education. Sir Wilson Jameson directed the campaign and he used every available channel of communication. The B.B.C. played a part, so did all the newspapers and magazines, and you may recall the dramatic posters with one simple slogan " Diphtheria Costs Lives—Immunization Costs Nothing".

How much further are we going to get with the control of whooping-cough and of outbreaks of food-poisoning if we fail to regard them as exercises in the medical education of the public? Here again we have reached a point at which the application of the knowledge now available must depend on its being impressed on the public.

### TUBERCULOSIS AND CANCER

Some similar considerations apply to tuberculosis and cancer. Early diagnosis is the key to each of these problems at the moment—the only key we have. How do we persuade the ordinary citizen of the importance of early diagnosis unless we give him all the information we can about early signs and symptoms and about the success of modern methods of treatment—provided they are applied at a sufficiently early stage? Here I would stress that the approach should be based upon hope rather than upon fear, and with the emphasis on curability.

Personally, I hold that we must undertake the medical education of the public. I would go further and say that this kind of medical education offers more hope of getting across health education than any other approach which is open to us.

Now look at the next question. How do we tell the public?

### HOW TO TELL THE PUBLIC

Here I think the individual doctor, the individual general practitioner, is the right person to tackle one important part of the problem. The National Health Service has so far been preoccupied with curative medicine largely to the exclusion of the preventive side. Yet the general practitioner is in an especially favourable position to further this kind of health education, since the patient is consulting someone whom he already knows and trusts and whose advice he will readily accept. The fact that advice is given at a time of temporary ill health makes it all the more likely that it will leave a lasting impression.

Any patient just recovering from an illness is very keen to know about that illness and how he got it and why, and how to avoid it in future. This proposal, unlike so many others, does not call for any major alteration in the curriculum. It is rather an orientation throughout the curriculum. We have to say to medical students: This is your medical education—but you will have to convey parts of it to your patients. Can you do so?

Now conveying medical facts and opinions to a layman is not easy. He has no scientific background as a rule and no knowledge of much of the language we use daily. As against this we can assume that he reads the papers, looks at some magazines, listens to the B.B.C. and looks at television. So he may have learnt a little, and we can certainly learn from studying how these mass media present medical facts and opinions.

The B.B.C. does a fair amount of simple health education in its programmes for schools, and here too there are regular broadcasts on science and biology. In sound broadcasting there have been a number of outstanding feature programmes, produced as a rule by Nesta Pain. On television, Mrs. Mary Vyvyan Adams and Philip Miller-Jones were responsible for the series called " Matters of Life and Death ". They were fortunate in having the guidance of Sir Henry Dale and the co-operation of a number of distinguished medical men. Quite recently the television people spent an hour on electro-encephalography at Dr. Grey Walter's laboratories and put over a first-class documentary feature.

The B.B.C., whether on sound or television, is always mindful of the enormous public it reaches. In all its major features it has taken the approach I have emphasized. " Matters of Life and Death " ranged over a number of major diseases—which was what the public wanted, and emphasized the importance of early diagnosis, the possibility of cure, and where relevant the modes of prevention—which is the information that we want to get across as a profession.

### THE PRESS

The daily newspapers and the large-circulation weekly magazines all take the same view. Health has no news value. Doctors and diseases and the ways they treat them are always in the news. You cannot pick up any newspaper on Friday without finding one, two, or three quotations from the *B.M.J.* and the *Lancet.* You can seldom pick up a paper on the 28th of any month without seeing *Family Doctor* widely quoted.

The main interest of the daily and Sunday papers is in medicine as news. And news is especially related to sex, crime, and dramatic operations. You can check this by searching your memory for the biggest medical stories of the last twelve months. They were concerned with Dr. Kinsey's latest work *Sexual Behaviour in the Human Female;* the Christie case; Professor Ian Aird and Baby Boko; and for full value on the operative approach to sex there was the story of Robert or Roberta Cowell.

Medicine makes news very frequently and that news is treated as such. But all the time the Press pays attention to doctors and to medicine and to medical features and stories. If you read critically the medical pieces in the dailies and weeklies you can learn a lot about the information that people want—and the way in which they want it served up. It would be pleasant if they could all reach and read the *B.M.J.* but they can't and they won't. They want a simplified version. They want interpretation.

### DOCTORS WRITING FOR THE LAYMAN

I think every doctor has a part to play in telling the public about medicine and especially the individual patient. But there is a special need for doctors who can write medical articles readably. There are very few doctors indeed who can write in a style acceptable to the public at large. Why that should be so I do not know—but it is so.

There are, of course, very real difficulties in presentation. In the modern world there is a fabulous proliferation of experts and specialists. In medicine and indeed in every other subject they are all addicted to jargon. It is all highly technical, highly scientific and regrettably obscurantist. The message may be admirable but it usually squelches along in a bog of jargon which might have been devised to achieve the maximum of words and the minimum of intelligibility.

Let us assume that the experts can communicate with each other, with a certain amount of semantic confusion it is true. But nothing that cannot be remedied by a couple of dozen letters in the Correspondence Columns of the *B.M.J.* and the *Lancet.* Now ask them to communicate with the ordinary layman. And mostly they cannot. They may try a turgid stilted writing-down style. But nobody—except an editor whose duty it is—would ever read it.

The prime error of the expert writing down is in his judgment of his audience. It is all too easy to go on using jargon which is then clumsily defined by other words which again are jargon. This criticism of the experts applies particularly when they try to write for a mass audience. The expert tends to assume what he regards as a basic level of education. His base, and he is leaning over backwards and contorting his own language to get down to that level, is still way above the heads of his audience.

Let me give you a classic example of this difficulty of communicating with a non-expert audience. It is taken from the annals of Proctor and Gamble, the largest makers of soaps and detergents in America. (*Time* 5th October, 1953, pp. 38–42) They have a big market research organization and they tested out on a very large sample of women the effect of some of their advertising.

They had in mind the use of the traditional prescription symbol—the ℞ for recipe or the invocation to the sun-god Ra. It is a symbol that is used very frequently in advertising because it suggests that the doctor has ordered it and therefore it must be good. They found that 40 per cent. of the women they interviewed had no idea what it meant. For them it had no meaning.

Then they were going to use the word " concentrated " in an advertisement. They tried that out and found that many housewives thought that it meant " blessed by the Pope ".

There are very few lay words which are not open to this kind of misinterpretation. That is why we have technical words. Yet in writing about health and about medicine it is essential to avoid technical jargon and it is not easy to choose words which can be understood, which are accurate, and which will bear only one meaning.

It is very salutary to take a letter in the *B.M.J.* or the *Lancet* and convert it into what one might call, without disrespect, *Daily Mirror* English. The *Mirror* is a readable paper and immensely popular. It has flourished on a series of very simple policy decisions. One of the important ones is to write it so that everybody can understand it and so that there can be no mistake about the meaning.

It is very difficult to simplify medical facts and opinions and preserve accuracy and authority at that particular level of clarity. But it can be done. I do not suggest that the *Mirror* style is ideal. But it does suit a readership of well over four million people. There will be more patients in the *Mirror* readership than in *The Times* group. And the same arguments apply to the *Express*, the *Mail*, the *Herald* and the *News Chronicle*. They are bought and read by millions. They all present facts and opinions at a level, and in terms, which suit millions of people.

### FAMILY DOCTOR

*Family Doctor* is well above the *Mirror* level of presentation but is deliberately written and presented to appeal to a broader section of the community than *The Times*. By and large it has the support of the profession and in it we aim at presenting readably, yet accurately and authoritatively, the information people want about health and disease, and about all those medical matters which are of absorbing interest to the public.

Looking ahead, I would put it like this. As a profession we can no longer neglect the public demand for information about our work. If we meet that demand we shall have better informed and more co-operative patients with a greater awareness of what we are trying to do for them. But in order to meet it we need an emphasis on telling the public right through all our medical teaching. We also need many more doctors with a special skill in writing and talking and lecturing and broadcasting about medicine.

To carry out this work effectively we will need to look again at our ethical rules, which have varied hardly at all in a time full of changes in every other direction. I think we need also to look more scientifically at our means and methods of medical and health education for the public. We ought to apply to them some of the yardsticks developed by social scientists and by the social-survey people.

As a profession we are only just moving into the field. *Family Doctor* is the first attempt in this direction by the profession as a whole. It is a bold experiment and I believe an important one. We are trying to get across facts about health, about medicine, and about doctors. They have to be presented so attractively that people will buy the B.M.A.'s popular health magazine in preference to all the hundreds of other magazines displayed on the bookstalls.

We have learnt a lot in the years since *Family Doctor* was launched in April 1951. We have a great deal more to learn. But one thing has become apparent. Every week we receive hundreds of letters from the readers of *Family Doctor*. By now I

must have read literally thousands and thousands of them. And from them I have learnt this.

All over this country there are men and women, ordinary pleasant intelligent people, who want to know more and more about medicine. They want to know about the care of babies and toddlers, about the care of sickness in the home, about the special care of old people. They want to know a great deal about individual diseases and disorders that have afflicted themselves or their relatives or their families.

This is not a morbid or deplorable curiosity. It is a natural and human wish for more knowledge about something that concerns them closely. In my view this is a wish that we should meet. A need for knowledge that as a profession we should supply.

